# Editorial of Special Issue “New Aspects of the Hepatocyte Growth Factor/c-Met System”

**DOI:** 10.3390/biomedicines3020201

**Published:** 2015-06-05

**Authors:** Giulia Ricci

**Affiliations:** Department of Experimental Medicine, Second University of Naples, Naples, Italy; E-Mail: Giulia.RICCI@unina2.it

HGF/c-Met system has covered, in the past two decades, multiple areas of research, from basic cell biology to clinical trials. This research topic witnessed a good deal of scientific interest and progress. These two molecules, in fact, have been discovered as being crucial elements in mediating multiple biological activities such as cell proliferation, survival, motility, morphogenesis and angiogenesis. It has been clearly demonstrated that these pleiotropic and complex biological functions act together during organogenesis and tissue homeostasis; however it can give rise to a broad spectrum of pathologies too, when the HGF/c-Met system is disrupted. Since I consider this topic of wide scientific interest, I felt the need to dedicate a special focus on this particular subject. The articles in this Special Issue are a collection of primary research papers and syntheses that have the merit to illustrate the most topical aspects of the HGF/cMet system biological functions. The goal is to provide a state-of-the-art overview, emphasize the new emerging paradigms and identify the current challenges to further scientific progress.

In this short editorial, I wish just to highlight the most relevant “take-home-messages” of this project. One of the highly topical subjects addressed by several articles concerns the involvement of the HGF/c-Met system in the onset and progression of cancer. This Special Issue contains manuscripts reviewing c-Met-dependent malignant behavior of the cells: the adaptor proteins and kinases involved in this process, and the other tyrosine-kinase receptors that enhance c-Met activation and signaling. Moreover, current knowledge about microRNAs known to regulate c-Met expression in tumors is also reported: this particular subject represents, in my opinion, a new promising field of research for basic science, cancer therapy, and cancer diagnosis. Other authors, highlight another important aspect of cancer progression, *i.e*., the relevance of HGF as a modulator of cancer and non-cancerous cells in the neoplastic microenvironment. Interestingly a mathematical model that describes the epithelium-mesenchyme transition of cancer cells has also been reported. In this regard, it is worth highlighting that research areas, having been to date historically separated, are becoming more related with multidisciplinary research teams representing, in my opinion, the future of good science.

The role of the HGF/c-Met system in tissue homeostasis and repair represents another relevant topic that has been outlined in this issue. In particular it has been pointed out that the HGF/c-Met system is involved in muscle regeneration and function, protection from acute tissue injury, chronic fibrosis, and in neurodegenerative diseases. This has led to ongoing HGF-based clinical trials for the treatment of patients with amyotrophic lateral sclerosis and spinal cord injury. Since HGF/c-Met system is a well-known modulator of the harmonic morphogenesis during embryonic development, the ability of HGF to drive tissue repair represents, in my opinion, a crucial link of this system to the success of stem cell-based therapies.

Finally, this Special Issue provides basic scientists and clinicians with a comprehensive, up-to-date overview of the newly available biotechnologies and compounds with potential for preventing organ failure and treating cancer.

In closing, I wish to thank the other Guest Editors for their personal contributions in the organization of this issue. A special mention is due to Prof. Maria Prat, for her commitment and support, which has been crucial to the success of this Special Issue. Moreover, I wish to personally thank all the authors who contributed and collectively made this dedicated issue possible.

